# Redefining boundaries: unveiling the schism in teacher-parent perceptions of educational engagement

**DOI:** 10.3389/fpsyg.2025.1539049

**Published:** 2025-07-17

**Authors:** Yael Fisher, Shiran Baissberg

**Affiliations:** Educational Administration, Achva Academic College, Arugot, Israel

**Keywords:** parental involvement, teachers' and parents' perceptions, facet theory, school-parent partnership, parental engagement, demographic balance

## Abstract

**Introduction:**

This study explores perceptions of parental involvement among parents and teachers within the Israeli education system. The research focus is on presenting and comparing the perspectives of each group, to understand the dynamics between these two crucial stakeholders in children's education.

**Purpose/hypotheses:**

The aim of the study was to understand and compare parents' and teachers' perceptions of parental involvement in the Israeli education system. The hypothesis was that there would be differences in how these two groups view parental involvement. Examining these perceptions uncover potential areas of misalignment or conflict that might impact the effectiveness of parent-teacher collaboration.

**Materials and methods:**

The primary methodological approach employed in this study was Facet Theory, supplemented by conventional statistical analyses. The study participants comprised 215 teachers from various schools nationwide, teaching grades 1–12, and 215 parents with children in the same grade range. To collect data, participants were asked to complete an anonymous self-report parental involvement questionnaire in which they classified parental functions based on their degree of agreement, ranging from absolute disagreement to approval and desire for such activities. This approach allowed for a comprehensive examination of parents' and teachers' perspectives across various educational levels.

**Results:**

The findings of this study indicate that parents' and teachers' perceptions of parental involvement differ fundamentally. Teachers' current perceptions of parental involvement were consistent with views reported in the literature three decades ago or more. They do not desire parental involvement beyond what they deem “necessary” and are averse to interference in their professional practice. According to this study's results, teachers perceive parental involvement to include the following functions: controlling school processes, participating in “obligatory” activities inherent to the parental role, and “service provision” –when needed, and they prefer that parents do not exceed the boundaries of “service provider.” Conversely, the parents' perceptions of parental involvement aligned with more recent research, which advocates for balanced parental engagement. Accordingly, parents are viewed as partners in the educational process at school, and it is believed that collaborative efforts between parents and school staff lead to improved student academic achievements. The research findings provide evidence that parents' perceptions of parental involvement consist of supporting and supplementing school resources, supervising school processes, partnership in extracurricular pedagogical processes, and maintaining awareness of internal pedagogical processes. Moreover, evidence suggests that these components relate to two loci of control: the school and its environment and the parent alone.

**Discussion:**

The comparison between teachers' and parents' perceptions revealed significant differences; observing them can serve as a solid foundation for fostering dialogue between the two parties. This dialogue can lead to a mutual understanding and a clear, agreed-upon definition of parental involvement, paving the way for a collaborative approach between parents and teachers in the Israeli education system and ultimately promoting student success.

**Limitations:**

This study has several limitations that should be considered when interpreting the findings, including a parent sample with higher educational attainment (83.3% holding academic degrees vs. 27.3% nationally) and predominantly female participants in both parent (86%) and teacher (93.5%) groups, which may reflect natural patterns of educational engagement but could limit broader generalizability. The online distribution method made it challenging to calculate precise response rates, and data collection during the COVID-19 pandemic may have influenced participants' perspectives on parental involvement. The findings are primarily applicable to the Israeli educational context and may require adaptation when considered in other cultural settings.

## 1 Introduction and theoretical background

### 1.1 Parental involvement

Parental involvement in the education system is a topic that has been researched worldwide, yet researchers cannot agree on a single, unambiguous definition (Fisher, 2018; Boonk et al., 2018). The literature presents diverse and complex areas of involvement, spanning a wide range of domains and actions that require definition, clarification, and understanding. Consequently, there is a need for a precise conceptualization and a clear definition of parental involvement in education (Yulianti et al., 2019; Epstein and Sheldon, 2023).

The research literature indicates a wide variety of approaches and definitions for the concept of parental involvement, addressing different areas: parental involvement in the child's life, partnership between parents and school, the school-student relationship, and various types of parental engagement (Park et al., 2017). Some approaches emphasize the teachers' perspective, limiting the parents' role to involvement in learning processes at home, after school hours (Woltran, 2023). Others emphasize that the parents' role in their child's academic success manifests through the numerous aspects of the parent-school relationship (Kantova, 2024; Boonk et al., 2018; Hornby and Blackwell, 2018).

Despite researchers' different approaches and definitions, three central axes can be identified in the field of parental involvement (Ma et al., 2016):

1. The system affected by the parent's involvement: Involvement in the home and/or after-school—i.e., the parents' involvement consists of creating a supportive learning atmosphere and helping with homework, and/or being active on school websites, in after-school activities, or in extracurricular events (Hamlin and Flessa, 2018). Involvement affecting the school consists of volunteering to participate in school activities. Parental involvement can also consist of a combination of the two systems.
2. Initiator of involvement: A distinction is made between contact initiated by parents (parents as proactive) and contact initiated by teachers (teachers as proactive, whereby parents are viewed as passive agents) (Fisher, 2011; Ðurišić and Bunijevac, 2017).
3. Purpose of involvement: Whether parental involvement is intended to contribute to or immediately change the student's academic, educational, emotional, or social domain (Piskorz-Ryń and Chikwe, 2024; Garbacz et al., 2018).

Many have attempted to define the concept based on the level of connection, level of involvement, and place of involvement. This ranges from basic communication between the school and parents reporting on the individual child to a more complex relationships between the school and parents that are not necessarily related to the individual child but to general volunteering in the school and community. Parents, teaching staff, and administration members create a connection at this level without addressing specific pedagogical or emotional issues. Involvement can be active, passive, or both active and passive (Fisher and Friedman, 2022; Povey et al., 2016; Park et al., 2017).

The prevailing approach among parental involvement researchers is that there is a need to establish a balance between the needs of parents and those of the school (Epstein and Sheldon, 2023). Research literature also emphasizes the main factors influencing parental involvement, such as their level of awareness and identification with the school. “Awareness” in this context refers to parents' cognizance of what is happening at school; i.e., rather than indifference to school activities, the parents show an active interest in what happens within the school and consider its contribution to their children's education as valuable. “Identification” in this context refers to the parents' acceptance of the school's values and goals. The degree of parental identification ranges from high identification, whereby parents approve of and support the norms and values that the school imparts to their children, which manifests as respect and trust between teachers and parents, to low identification, meaning that parents do not approve of the norms and values that the school imparts to their children (Yulianti et al., 2019).

Additionally, the approach espoused in the research literature is to recognizes the authority of both the parents and the school, demonstrating that this allows for positive parental involvement (Ðurišić and Bunijevac, 2017; Hornby and Blackwell, 2018).

### 1.2 Factors influencing parental involvement in education

Parental involvement is widely recognized as a significant contributor to students' academic and emotional success in school. Research consistently demonstrates the positive impact of parental involvement, particularly on students' academic achievements (Martinez-González et al., 2023). However, parental involvement levels demonstrate a normal distribution pattern, ranging from minimal to extensive engagement (Fisher and Kostelitz, 2015).

The child's age emerges as a significant determinant of parental involvement factors. Elementary school children generally welcome parental involvement, whereas adolescents tend to seek greater autonomy and may resist parental engagement. Parents similarly report higher motivation to assist younger children, often feeling more competent with elementary-level content than with secondary school material (Wei et al., 2019).

In examining influential parent- and family-related factors, the spectrum ranges from socioeconomic status to educational encouragement at home. Parental involvement in Western societies correlates with educational attainment levels, democratization processes, educational competition, and student feedback systems (Foulidi and Papakitsos, 2022; Chen et al., 2024). Gender differences persist, with mothers generally showing higher engagement levels than fathers (Kim, 2022). Socioeconomic status significantly impacts involvement patterns: parents from lower socioeconomic backgrounds typically delegate greater educational responsibility to the school. In comparison, parents of a higher socioeconomic status demonstrate greater participation in school governance and activities (Zhang et al., 2023).

Parent-teacher relationship factors play a crucial role, with school initiatives and invitations for involvement often proving more influential than socioeconomic factors (Tauber et al., 2023). Modifiable factors, such as communication quality, school encouragement, and engagement opportunities, significantly impact parental involvement levels (Epstein and Sheldon, 2023). The relationship between parents and school staff is a fundamental determinant of engagement levels, with increased opportunities for involvement generally leading to higher participation rates. Limited legislative frameworks, insufficient resource allocation, and inadequate teacher training in parental engagement influence the extent and quality of parental involvement across different educational contexts (Yang et al., 2023). Research has identified significant correlations between public region employment, increased school involvement, and higher engagement rates among parents with multiple children in the same school (Mori, 2022; Fisher and Kostelitz, 2015). This relationship between community service orientation and school involvement suggests broader implications for understanding parental engagement patterns. The connection between parental involvement and community volunteering reveals interesting patterns, particularly regarding collective engagement (Fisher, 2018).

### 1.3 Models of parental involvement in education

Models of parental involvement span a broad spectrum, ranging from hierarchical approaches to models advocating full parental partnership in all institutional education matters. Earlier research, particularly before 1994, primarily focused on traditional school activities, such as parent-teacher conferences, participation in school fundraising, school fairs, bake sales, and similar events (Goldberger, 1996). These models predominantly emphasized a hierarchical approach to parental involvement: from less desired to most desired forms of involvement. Such models highlighted that power resided with the school, positioning it as the sole entity authorized to create policies.

Parental involvement models have evolved significantly over the past few decades, with five significant frameworks emerging as particularly influential in educational research and practice. These models provide different yet complementary perspectives on how and why parents engage in their children's education.

Epstein's (2019) Framework of Six Types of Involvement is one of the most comprehensive and widely adopted models proposed in educational research. Initially developed by Joyce Epstein in 1995 and updated through multiple editions, with the most recent comprehensive Handbook published in 2019, this model provides a holistic approach to understanding parental involvement. The framework encompasses parenting, communicating, volunteering, learning at home, decision-making, and community collaboration. Recent research by Park et al. (2017) has validated the continuing relevance of this model in contemporary educational settings, particularly emphasizing its applicability across diverse cultural contexts.

The Hoover-Dempsey and Sandler Model offers a psychological perspective on parental involvement (Hoover-Dempsey and Sandler, 1997). This model is particularly valuable for understanding the motivational aspects of parental engagement and has been instrumental in developing intervention strategies to enhance parent participation. This model has been refined by Hoover-Dempsey et al. (2010). Their contemporary research explores the psychological factors influencing parents' decisions to become involved in their children's education.

Contemporary research indicates that the most effective approaches to parental involvement often integrate elements from multiple models. As the study of Park et al. (2017) demonstrated, successful parental involvement programs typically draw on various frameworks to create comprehensive approaches tailored to specific school contexts. This integration acknowledges the complexity of parent-school relationships and the need for flexible, context-sensitive approaches to foster meaningful parental involvement.

The collective influence of these models has significantly shaped our understanding of how schools can effectively engage parents in their children's education. While each model offers unique insights, they share a common recognition of the multifaceted nature of parental involvement, the importance of two-way communication, the need to address barriers to involvement, and a focus on improving student outcomes. As educational contexts evolve, these models provide valuable frameworks for understanding and promoting effective parental involvement in education.

### 1.4 Locus of control

The use of the term “locus of control” in the context of parental involvement is novel, although it is well-known in other fields. The term originated from Rotter's (1954) social learning theory of personality; a few years later, Lefcourt (1966) referred to “perceived control” and defined it as follows: “Perceived control is …a generalized expectation for internal versus external control of reinforcement.” Rotter (1975) clarified that internal and external locus of control do not refer to dichotomous states but represent two ends of a continuum.

While some individuals attribute an internal locus of control to outcomes or events, others attribute the control of such events to external circumstances. For this study, the term “locus of control” was borrowed to describe the degree to which parents attribute their involvement in their children's educational processes to personal internal factors or to external school-related circumstances. Like Rotter's articles, this study does not view the factors as dichotomous but as endpoints on a spectrum.

Hence, in this context, the two ends of the locus of control continuum are factors related to the parents and, at the other end, factors related to the school and its environment. *Parental Locus of Control:* Actions or activities indicative of parental involvement depend entirely on the parents. Examples of these actions include choosing the school the child attends, helping with homework, accompanying the class on a field trip, and attending parent-teacher meetings. *School's Locus of Control:* Parental involvement can occur only with the school's (and its environment's) consent. The parent cannot perform these actions without the school's cooperation and willingness to accept the parent as an active participant. Examples of these actions include visiting the school during the day, conducting activities for the class or the entire school, and developing curricular programs.

## 2 Materials and methods

The primary objective of this research was to empirically examine the theoretical structure of parental involvement perceptions from two distinct perspectives, those of teachers and those of non-teacher parents, and to analyze the differences between these perspectives.

Facet theory (Guttman, 1959) was the methodological approach chosen for this purpose, as it provides a comprehensive framework for examining complex, multifaceted phenomena in social and behavioral sciences. This approach operates on the premise that complex phenomena can be systematically deconstructed into interrelated components or “facets,” each contributing to a holistic understanding of the subject under investigation (Levy, 2005). Rather than isolating individual variables, Facet Theory emphasizes the examination of multiple dimensions simultaneously, acknowledging their interdependent nature.

The theoretical underpinning of this methodology rests on the assumption that social and behavioral phenomena possess inherent structural properties that can be identified and analyzed through careful examination of their constituent facets (Shye et al., 1994). Facet Theory enables researchers to construct a more nuanced and complete understanding of complex phenomena by providing a systematic framework for identifying, measuring, and analyzing these multiple dimensions.

At its core, this approach facilitates the integration of multiple conceptual elements into a coherent analytical framework, allowing researchers to examine how different aspects of a phenomenon interact and influence each other. This integrative approach moves beyond traditional linear analysis to capture the multidimensional nature of social and behavioral constructs. The strength of Facet Theory lies in its ability to bridge the gap between theoretical conceptualization and empirical investigation. It provides researchers with tools to examine complex relationships while maintaining theoretical coherence systematically (Levy, 2005).

The primary tool in Facet Theory is the “mapping sentence.” This sentence is a foundation for hypotheses regarding empirical relationships between research variables and their geometric spatial distribution, hence termed “structural hypotheses.” The function of the mapping sentence is analogous to that of the hypotheses formulated in conventional statistical methodologies

**Figure 1:**
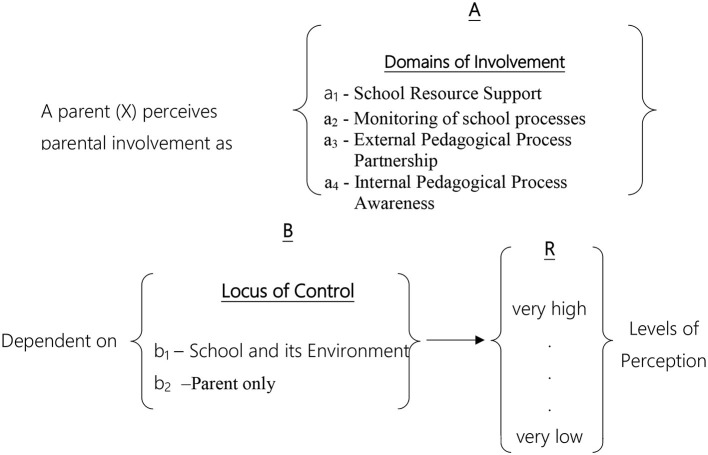
Mapping sentence for observations of parents' perceptions of parental involvement.

**Figure 2:**
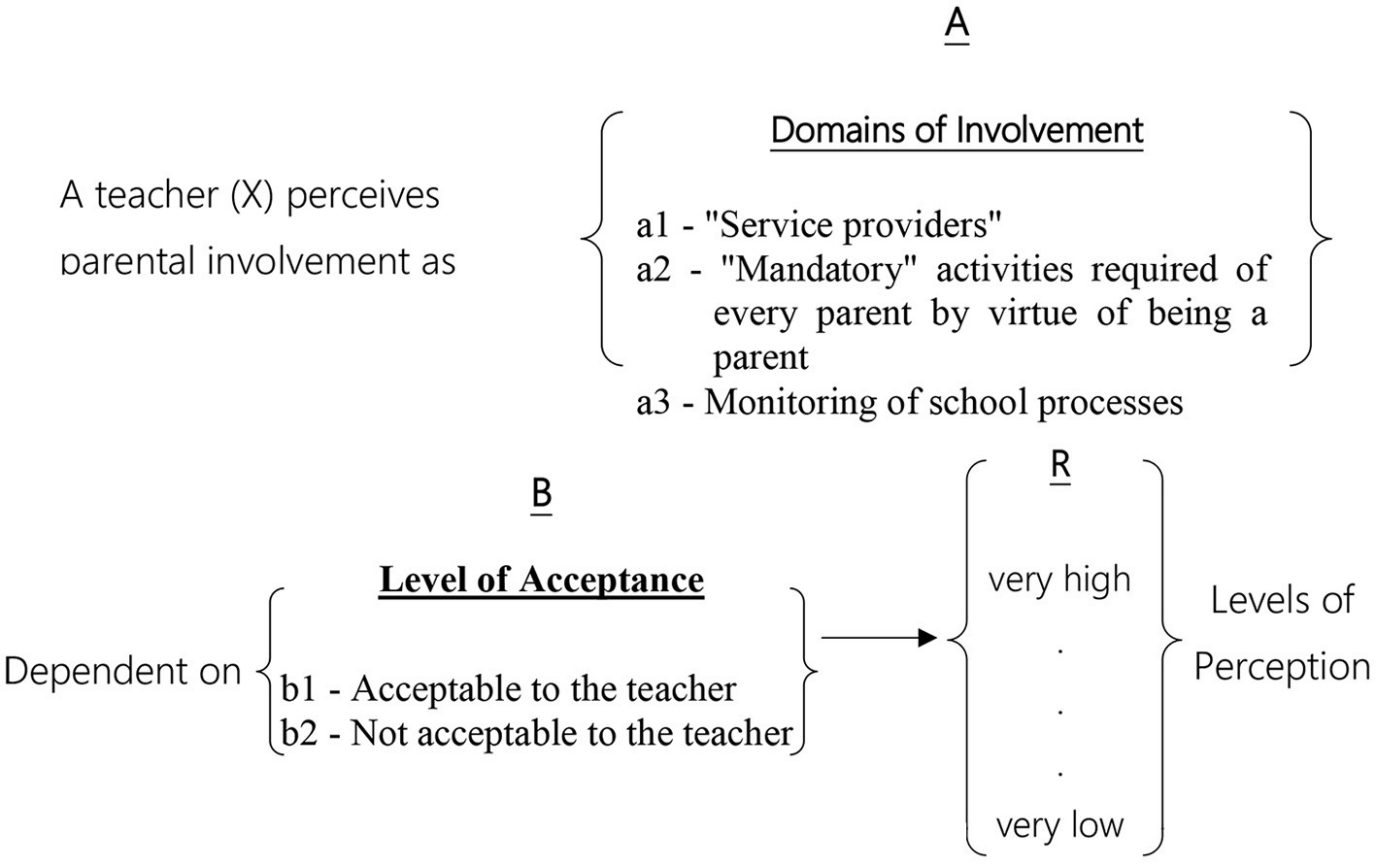
Mapping sentence for observations for teachers' perceptions of parental involvement.

In most studies employing facet theory methodology, a single mapping sentence is formulated and designed to address hypotheses regarding a single population. However, as this study aimed to compare two populations, two distinct mapping sentences were developed. The first pertains to the parent population (see Figure 1), while the second addresses the teacher population (see Figure 2).

### 2.1 Definition of variables and research hypotheses

#### 2.1.1 Parents' perceptions

Parents' perception of parental involvement can be classified according to two content facets:

A– Areas of Involvement: Parents define parental involvement as focusing on four main domains. The components in this Facet are not structured; therefore, this Facet will distribute the variables in the SSA map in a polarizing structure. This Facet contains four regions:
  a_1_. *Support of School Resources*- These areas focus on matters related to parental support of the school system, where parents “assist” the school in various aspects, such as volunteering for the class committee or collecting and raising funds for school activities.
  a_2_. *Monitoring School Processes*- In these areas, parents actively monitor processes occurring within the school and even participate in decision-making on various issues, such as providing feedback on the performance of teachers and administration and conducting structured school visits.
  a_3_. *Partnership in Extracurricular Pedagogical Processes*- In these areas, parents take an active role in all pedagogy-related matters outside the school concerning their children, such as reviewing notebooks and preparing for exams.
  a_4_. *Awareness*^1^ *of Internal School Pedagogical Processes*- These areas relate to parents' awareness regarding school activities, such as attending parent-teacher meetings, knowing the teaching staff, and being informed about activities taking place at school.
B– Locus of Control: The locus of control facet divides the areas of involvement into two regions, and its components can be positioned along a continuum (axis). Therefore, this Facet will distribute the variables in the SSA map in an axial structure.
  b_1_. *Locus of Control Resides within the School and its Environment*- These activity areas relate to matters that do not depend solely on the parent. For the parent to participate or perform specific actions, the decision-making process is not theirs alone but depends on the school or factors in the school environment. For example, a parent cannot independently decide to participate in pedagogical committees, as this requires obtaining permission from the school and staff.
  b_2_. *Locus of Control Resides with the Parent Alone*- These activity areas relate to decisions that depend solely on the parent; only the parent can decide whether to participate in or abstain from certain activities. For instance, it is solely the parents' decision whether to accompany the child's class on a field trip; similarly, the parents alone can choose which school they wish their child to attend.

#### 2.1.2 Teachers' perceptions

Teachers' perception of parental involvement can be classified according to two content facets:

A– Areas of Involvement: Teachers define parental involvement as focusing on three main domains. The components in this Facet are not structured; therefore, this Facet distributes the variables in the SSA map in a polarizing structure. This Facet contains three regions:
  a_1_. “Service Providers” - These areas relate to all matters in which parents “assist” by providing a service to the school, such as helping set up class parties and being a member of the parents' committee.
  a_2_. “Mandatory” Activities for Every Parent by Virtue of Being a Parent - These areas relate to activities that parents must perform as part of their parental role, such as helping with homework and becoming acquainted with the school staff.
  a_3_. Control of School Processes - These areas focus on matters related to parents' desire to supervise the school system. Parents actively monitor and participate in managerial and pedagogical processes, such as participating in curriculum development and teacher hiring and dismissal.
B– Degree of Teacher Approval: The degree of teacher approval facet divides the areas of involvement into two regions. The components in this Facet are positioned along a continuum (axis); therefore, this Facet will distribute the variables in the SSA map in an axial structure.
  b_1_. Acceptable to Teachers - These areas relate to matters where teachers agree to parental involvement, primarily due to the understanding that parents have the right to take some part in school life or due to the tangible assistance they provide in these areas. Examples include physical aid at school and help with homework.
  b_2_. Unacceptable to Teachers - These areas relate to matters in which teachers would prefer that the parents not get involved. Teachers disapprove of parental involvement in these areas and would prefer that these issues be addressed solely by the professional and educational staff. Examples include grade appeals and awareness of the class's overall achievements.

### 2.2 The study population

The sample consisted of approximately 430 participants, including 215 parents (who were not working as teachers in the education system) and 215 teachers.

#### 2.2.1 Parent group demographics

Parents were recruited through multiple official online channels to ensure broad representation:

a. School-based recruitment through official parent communication systems (school websites, parent portals, newsletters).
b. The parent committee networks across different school types and regions.
c. Community centers and local authority family services departments.
d. Social media groups specific to different geographic regions and types of schools.
e. Parent education organizations and support groups.

Recognition that online recruitment may systematically exclude specific populations (lower socioeconomic status, older participants, those with limited digital literacy) led to supplementary recruitment through community centers offering computer access and assistance. Additionally, the survey was optimized for mobile devices to increase accessibility across different technological capabilities.

The parent group comprised 86% (185) women and 14% (30) men. Age distribution showed 46% (99) were 50+, 43.3% (93) were 40–49, and 9.8% were 30–39. Two respondents did not specify their age. Regarding education, 47.4% (102) held a bachelor's degree, 32.1% (69) held a master's degree, 3.7% (8) held a doctoral degree, and 16.7% (36) reported other educational qualifications. Employment status showed that 80.9% (174) were salaried employees, 13% (28) were self-employed, 1.4% (3) were seeking employment, and 4.7% (10) were unemployed. Family size distribution indicated that 46.5% (100) had three children, 26.5% (57) had two children, 20% (43) had four children, 3.7% (8) had one child, and 3.3% (7) had five children or more. Regarding children's ages, 36.7% (79) of the cohort had children of ages 6–9, 34.9% (75) of the parents had children of ages 13–18, and 28.4% (61) had children of ages 10–12. Residential distribution showed 54.9% (118) lived in urban areas, 40.9% (88) in rural communities or community settlements, and 4.2% (9) in kibbutzim. Among the respondents, 18.1% (39) had a child identified as gifted, while 81.4% (176) did not. Regarding school type, 62.3% (134) had a child enrolled in a secular state-run elementary school (grades 1–6 or 1–8), 4.2% (9) had a child enrolled in a religious state-run elementary school, 30.6% (66) had a child attending a secular state-run secondary school (with 6 years, grades 7–12) or a high-school (grades 10–12), and 2.8% (6) had a child enrolled in a religious state-run secondary school.

#### 2.2.2 Teacher group demographics

Teachers' recruitment employed parallel online channels to mirror teacher demographics:

a. Ministry of Education regional offices distributed invitations through official communication channels.
b. Teacher union organizations shared the survey through their member networks
c. Professional development platforms and educational forums posted recruitment notices
d. School principals were contacted to forward invitations to their teaching staff
e. Educational conference organizers shared the survey with attendees

The teacher group consisted of 93.5% (201) women and 6% (13) men, with one of the respondents (0.5%) not specifying gender. Age distribution showed that 40.9% (88) were ages 41–55, 33% (71) were ages 31–40, 17.2% (37) were ages 20–30, and 8.8% (19) were 55 or over. In terms of educational achievements, 50.7% (109) reported having a bachelor's degree, 47.9% (103) had a master's degree, 0.9% (2) reported other qualifications, and 0.5% (1) held a doctoral degree. Regarding school size, 65.7% (139) taught in schools with 400–700 students, 26% (56) in schools with 700–1,000 students, and 9.3% (20) in schools with over 1,000 students. School type distribution showed that 67.5% (145) taught in secular state-run elementary schools, 7% (15) in religious state-run elementary schools, 21.9% (47) in secular state-run secondary schools, and 3.8% (8) in religious state-run secondary schools. Teaching experience indicated that 49.8% (107) had over 10 years experience, 23.3% (50) had 6–10 years, 22.8% (49) had 2–5 years, and 4.2% were in their first year. Regarding roles, 42.8% (92) were homeroom teachers with additional responsibilities, 29.3% (63) were homeroom teachers only, 27.4% (59) taught a specific subject, and 0.5% (one respondent) were a school principal. Additionally, 68.4% (147) indicated they were not members of the school management, while 31.6% were management team members.

### 2.3 The research instruments

The researchers employed an anonymous self-report parental involvement questionnaire titled “Questionnaire for Measuring Attitudes Toward Parent-School Relations” (Fisher, 2011). The questionnaire consisted of two sections and included an introductory letter to participating teachers and parents outlining the study's aims and objectives, providing assurance of anonymity, and soliciting their candid participation.

The first section comprises 44 statements forming the Parental Perception of Involvement Scale (PPIS) (α = 0.91). Response options range from 1 (strongly disagree) to 5 (strongly agree). The original questionnaire yielded four factors: enhancement of school resources, monitoring of school processes, pedagogy, and school welfare. The reliability coefficients for these four factors were 0.80, 0.85, 0.92, and 0.70, respectively.

The second section included demographic variables (nine items common to parents and teachers: e.g., gender, age, education). The teacher questionnaire included additional background variables: number of students in their school, school type, teaching experience, role in school, and management team membership. The parent questionnaire included additional variables: employment status, number of children, children's ages, type of residential area, whether their child was identified as gifted, and type of school their child attended.

### 2.4 Data collection and analysis

The study was conducted during the 2020–2021 academic year. The research was conducted in three main phases. The first phase included submitting the original questionnaire and the research proposal to the relevant college's ethical committee (removed for blinded submission) for approval. In the second phase, questionnaires were administered to 215 parents (residing in various areas with children attending different schools) and 215 teachers (from multiple types of schools and locations). The third phase involved data processing, beginning with Confirmatory Factor Analysis (CFA) using SPSS 21, followed by data analysis using HUDAP 8 (Hebrew University Data Analysis Package Version 8).

### 2.5 Ethical considerations and privacy protection

Participant anonymity was strictly maintained throughout the data collection process, with no collection of personal identifiers (such as names, contact information, or addresses). Participation was entirely voluntary, and teachers could exercise their right to withdraw by either declining to complete the questionnaire or submitting an incomplete form. Any incomplete questionnaires were destroyed through secure shredding procedures, and the corresponding data were excluded from the final analysis. The study was conducted without monetary compensation for participation, and participants incurred no costs associated with their involvement in the research.

## 3 Results

### 3.1 Factor analysis

The data analysis in this research followed a two-phase approach. In the first phase, factor analysis was conducted to obtain a comprehensive overview of the questionnaire items. While factor analysis is not always employed in Facet Theory studies, it proved beneficial for the initial stage, based on similar research precedents. The original parental involvement scale (Fisher, 2011) utilized item-total correlation analysis.

On the Parents' Perceptions of Parental Involvement Scale (PPPIS), the Cronbach's alpha coefficient was α = 0.93 (44 items). In this scale, there was no need to eliminate items. Although Confirmatory Factor Analysis (CFA) revealed four factors as in the original scale (Fisher, 2011), the component structure differed, leading to the decision to conduct an Exploratory Factor Analysis (EFA). After EFA, four factors emerged:

1. CSP - Control of School Processes (15 items; α = 0.91)
2. SSR - Support of School Resources (14 items; α = 0.91)
3. AISPP - Awareness of Internal School Pedagogical Processes (9 items; α = 0.87)
4. PISPP- Participation in Internal School Pedagogical Processes (6 items; α = 0.82) (Table 1).

**Table 1:** Parents' perceptions of the concept of “parental involvement.”

**Item no**.	**Item content**	**Factor 1**	**Factor 2**	**Factor 3**	**Factor 4**
**Factor 1: control of school processes (15 items; Eigenvalue** = **11.82; explained variance** = **26.87%;** α = **0.91)**					
23	Hiring and firing administrators	0.848	0.020	−0.005	−0.129
22	Hiring and firing teachers	0.837	−0.013	0.021	−0.135
24	Teacher placement	0.830	−0.027	−0.017	−0.039
20	Curriculum development	0.714	0.337	0.048	0.061
21	Curriculum criticism	0.704	0.194	0.212	0.030
26	General teacher criticism	0.667	−0.055	0.236	0.036
9	Weekly school visits	0.623	0.245	−0.148	0.314
8	Classroom visits during the school day	0.599	0.180	−0.153	0.258
19	Participation in pedagogical committees	0.592	0.308	0.107	0.045
36	Partnership in decision-making	0.574	0.218	0.491	0.051
33	Awareness of student achievements	0.552	−0.030	0.232	0.243
35	Meetings with principal	0.522	0.310	0.425	−0.005
44	Weekly phone contact	0.489	0.170	0.070	0.241
10	Opinion on lesson quantity	0.436	0.133	0.007	0.166
25	Intervention in teacher behavior	0.398	0.018	0.357	0.003
**Factor 2: support of school resource (14 items; Eigenvalue** = **5.20; explained variance** = **11.82%;** α = **0.91)**					
5	Organizing fairs	0.131	0.802	0.005	0.075
1	Class committee	0.023	0.763	0.211	−0.071
6	Class parties	−0.100	0.734	0.048	0.223
18	Informal activities	0.237	0.728	0.218	0.085
11	Class lesson	0.193	0.703	0.095	0.079
12	School activity	0.322	0.696	0.079	−0.060
2	School committee	0.210	0.682	0.174	−0.171
14	Fundraising	0.117	0.677	0.188	0.046
17	Physical help	0.062	0.664	0.116	0.338
16	School fundraising	0.372	0.632	−0.052	0.017
4	Trip accompaniment	−0.136	0.558	0.120	0.174
13	New immigrant adoption	0.131	0.515	0.196	0.142
15	Program funding	0.358	0.467	0.021	0.274
3	Free choice of school	−0.009	0.301	0.073	0.060
**Factor 3: awareness of internal pedagogical processes (nine items; Eigenvalue** = **3.93; Explained Variance** = **8.93%;** α = **0.87)**					
29	Knowledge of activities	−0.035	0.193	0.792	0.177
30	Knowledge of curriculum	0.097	0.147	0.768	0.150
28	Knowledge of staff	−0.033	0.182	0.742	0.161
31	Knowledge of class population composition	0.067	0.141	0.676	0.185
34	Knowledge of staff decisions	0.330	0.164	0.661	0.169
32	Awareness of violence issues	−0.042	0.177	0.631	0.261
27	Knowledge of social relationships	0.250	0.189	0.575	0.234
7	Attendance at teacher-parent meetings	−0.324	0.298	0.372	0.244
**Factor 4: participation in internal pedagogical processes (nine items; Eigenvalue** = **2.17; explained variance** = **4.93%;** α = **0.82)**					
40	Test preparation	−0.053	0.218	0.231	0.813
37	Homework help	−0.169	0.194	0.194	0.758
38	Notebook checking	0.137	0.118	0.118	0.706
43	Test review	0.125	0.110	0.110	0.623
42	Involvement in grade appeals	0.296	0.089	0.089	0.521
39	Involvement in discipline issues	0.161	−0.064	−0.064	0.515
41	Supporting a child in disagreements with a teacher	0.276	−0.058	−0.058	0.422

Factor analysis was conducted to validate the first stage before data analysis using Facet Theory. Weighted Smallest Space Analysis (WSSA) was performed in the second data analysis stage using HUDAP (Hebrew University Data Analysis Program), building upon the factor analysis conducted in the first stage.

On the Teachers' Perceptions of Parental Involvement Scale (TPPIS), the overall Cronbach's alpha coefficient was α = 0.92 (44 items). Three items were eliminated from the original scale based on the following criteria: Corrected Item-total Correlation < 0.35 and factor loading below 0.35. Following item elimination, factor convergence was re-examined through Confirmatory Factor Analysis (CFA). The CFA did not validate the hypothesized structure, so an Exploratory Factor Analysis (EFA) was conducted. The EFA of the TPPIS yielded three factors (in contrast to the four factors identified in the original scale):

1. SV – “Service Providers” (14 items; α = 0.91)
2. CSP - Control of School Processes (13 items; α = 0.91)
3. MA – “Mandatory” Activities Inherent to Parental Role (14 items; α = 0.89) (Table 2)

**Table 2:** Teachers' perceptions of the concept of “parental involvement.”

**Item no**.	**Item content**	**Factor 1**	**Factor 2**	**Factor 3**
**Factor 1: “service providers” (14 items; Eigenvalue** = **10.56; explained variance** = **25.75;** α = **0.90)**				
5	Organizing fairs	0.803	−0.023	0.061
12	School activities	0.783	0.047	0.090
2	School Committee	0.763	−0.007	0.029
1	Class committee	0.757	−0.090	0.060
17	Physical assistance	0.755	0.066	0.224
14	Fundraising	0.745	0.068	0.035
6	Class parties	0.738	−0.101	0.226
11	Teaching a class	0.725	0.016	0.195
18	Informal activities	0.710	0.050	0.258
16	School Fundraising	0.674	0.157	−0.056
13	Supporting new immigrants	0.597	−0.037	0.138
4	Accompanying Field trip	0.575	−0.096	0.165
15	Program funding	0.571	0.239	0.092
3	Free choice of school	0.464	0.052	0.185
**Factor 2: control of school processes (13 items; Eigenvalue** = **6.98; explained variance** = **17.02;** α = **0.91)**				
22	Teacher hiring and dismissal	−0.141	0.873	0.052
23	Principal hiring and dismissal	−0.107	0.853	0.059
24	Teacher placement	−0.158	0.849	0.018
21	Curriculum criticism	0.013	0.780	0.150
26	General teacher criticism	−0.164	0.764	0.100
8	Classroom visits during the day	0.082	0.696	0.029
20	Curriculum development	0.226	0.684	0.118
25	Teacher behavior intervention	−0.045	0.683	0.233
10	Opinion on class quantity	0.131	0.648	−0.069
9	Weekly visits	0.105	0.633	0.159
33	Student achievement awareness	−0.004	0.574	0.263
19	Participation in pedagogical committees	0.246	0.519	0.072
36	Decision-making partnership	0.244	0.472	0.319
**Factor 3: “mandatory” parental activities (14 items; Eigenvalue** = **3.43; explained variance** = **8.36;** α = **0.89)**				
40	Test Preparation	0.142	−0.088	0.773
43	Test review	0.017	0.084	0.751
37	Homework assistance	0.217	−0.178	0.688
39	Discipline issues involvement	0.020	0.159	0.663
38	Notebook checking	0.035	0.020	0.657
29	Knowledge of activities	0.385	0.134	0.637
30	Knowledge of curriculum	0.284	0.238	0.606
32	Awareness of violence issues	0.312	0.162	0.570
27	Knowledge of social relationships	0.158	0.356	0.557
31	Knowledge of class demographics	0.171	0.267	0.536
28	Knowledge of staff	0.342	0.063	0.531
42	Grade appeals	−0.002	0.355	0.520
34	Knowledge of staff decisions	0.230	0.380	0.507

### 3.2 The Smallest Space Analysis maps

#### 3.2.1 The SSA map of parents' perception

The data distribution was initially examined in a two-dimensional representation (space represented by width and length). The Coefficient of Alienation found in the parents' map was 0.18, representing a good fit between the graphical representation of variables in their spatial distribution maps. The HUDAP software enables marking and calculating the spatial boundaries of items in facets as defined in the mapping sentence and plotting them in the spatial distribution map of variables.

The Separation Index (goodness of fit) of the data distribution of facets A and B in the two maps was 1.000.

**Figure 3:**
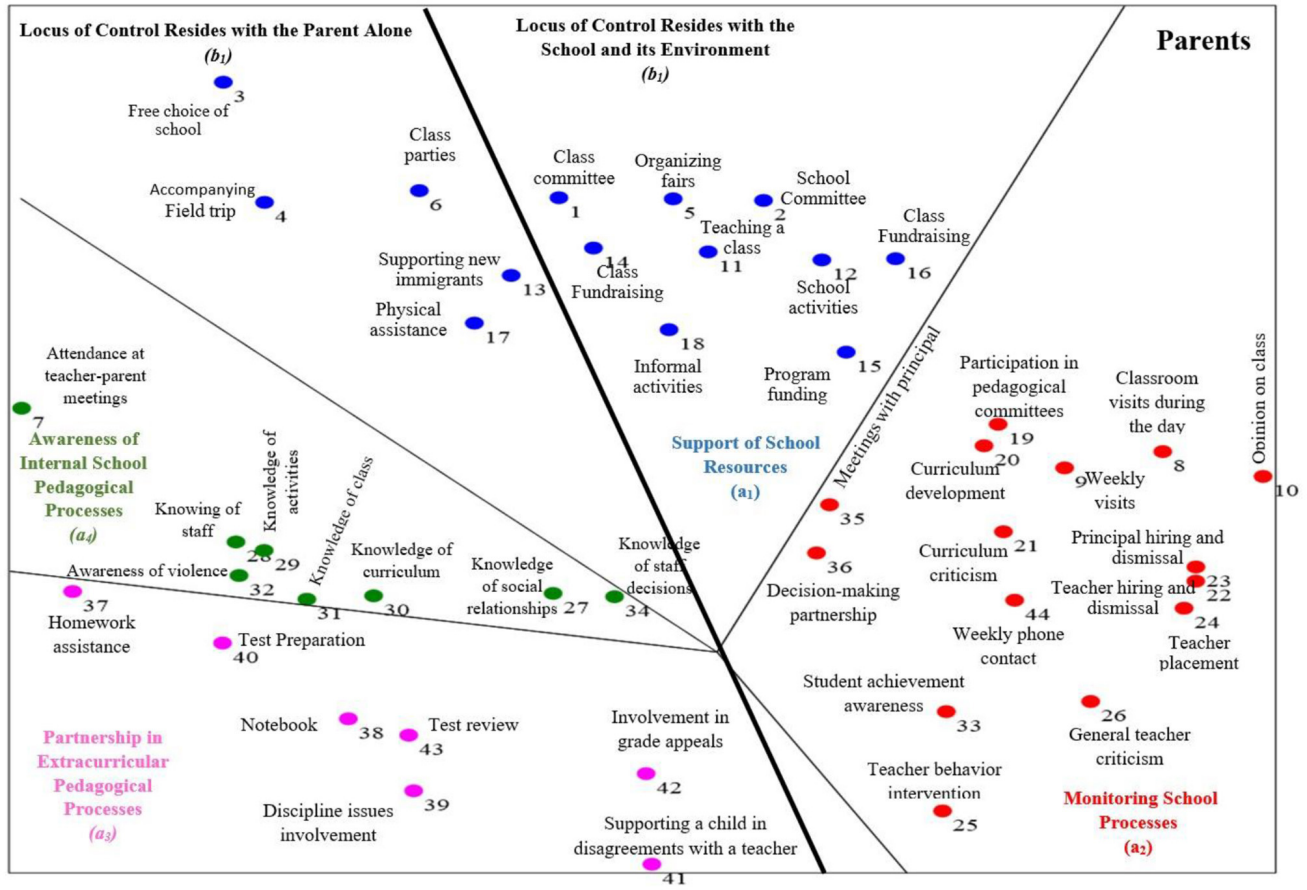
The integrated SSA map – parents' definition parental involvement.

The significance of the goodness of fit index indicates perfect correspondence between the items' locations on the obtained maps and the hypothesized locations according to the facets. Both indices obtained in data processing—the Coefficient of Alienation and the Separation Index—demonstrated that the data presented in the SSA maps (Figure 3) enable the highest-level inference regarding the structure of distribution and concept. The data distribution across the obtained SSA maps created a combined pattern of axial and polar distributions.

Facet A represents an angular data distribution. In this configuration, items are arranged in angular regions emanating from a single point in a fan-like formation. This type of distribution emerges when the items within the Facet are not classified according to any specific ranking or order. The function of this type of Facet is Polarizing, meaning that each item in the area corresponds to a different direction in the geometric space (Friedman, 2011). The organization of regions in the SSA map enables examining the relationships between the regions. Regions with similar semantic content are expected to demonstrate proximity, while regions with opposing semantic content are expected to show contrast. Observation of the map (Figure 3) reveals that it is divided into four angular regions. We will review them in a clockwise rotational movement, beginning from the upper central section (region a_1_).

The upper region contains 14 items expressing support for school resources (a_1_). This region represents areas where parents support school resources and “assist” in ongoing activities while also participating in the enrichment and expansion of school-provided activities (for example, “Teaching a lesson to a class”—item 11; “helping with class parties”—item 6).

The right region includes 15 items expressing Supervision of School Processes (a_2_). This region is characterized by domains related to parental monitoring of school processes (for example, “Expressing criticism about the curriculum to the management team”—item 21; “Intervention in inappropriate teacher behavior”—item 25).

The lower left region comprises seven items representing Partnership in Extracurricular Pedagogical Processes (a_3_). This region reflects processes in which parents take an active role in the school in pedagogical matters related to their children (for example, “help with test preparation”—item 40; “reviewing tests”—item 43).

The upper left region contains eight items representing awareness of internal pedagogical processes (a_4_). This region manifests predominantly as passive involvement, primarily centered on parents' Awareness of Internal School Pedagogical Processes (for example, “knowledge of school activities”—item 29; “familiarity with curricula”—item 30).

An axial facet analysis (Facet B) examined the variance in parental perceptions regarding Locus of Control. The axial deployment in the resulting SSA map divides the map into two sections—right and left—with items positioned continuously relative to their distance from the axis. All items are positioned along the axis in geometric space.

The region on the right comprises 24 items and represents the locus of control held by the school and its environment (b_1_). This region pertains to matters and processes “controlled” by the school and its environment, which parents cannot execute without the support or assistance of the school and its affiliated entities (e.g., “participating in pedagogical committees”—item 19; “funding projects”—item 15).

The left region contains 20 items and represents topics and processes relating to activities where the locus of control lies solely with the parents (b_1_). This region includes items of activities and processes that parents can independently decide whether to perform without depending on other factors (e.g., “attending parent-teacher meetings”—item 7; “familiarity with classroom social composition”—item 27).

#### 3.2.2 The SSA map of the teachers' perception

The data distribution was initially examined in a two-dimensional representation (space represented by width and length). The Coefficient of Alienation^2^ found in the parents' map was 0.18, representing a good fit between the graphical representation of variables in their spatial distribution maps. The HUDAP software enables marking and calculating the spatial boundaries of items in facets as defined in the mapping sentence and plotting them in the spatial distribution map of variables.

The Separation Index^3^: The goodness of fit (data distribution of facets A and B in the two maps) was 1.000.

The significance of the goodness of fit^4^: The index indicates perfect correspondence between the items' locations on the obtained maps and the hypothesized locations according to the facets. Both indices obtained in data processing—the Coefficient of Alienation and the Separation Index—demonstrated that the data presented in the SSA maps enable the highest inference level regarding the structure of distribution and concept. The data distribution across the obtained SSA maps creates a combined angular (axial) and polarizing (polar) distribution pattern.

**Figure 4:**
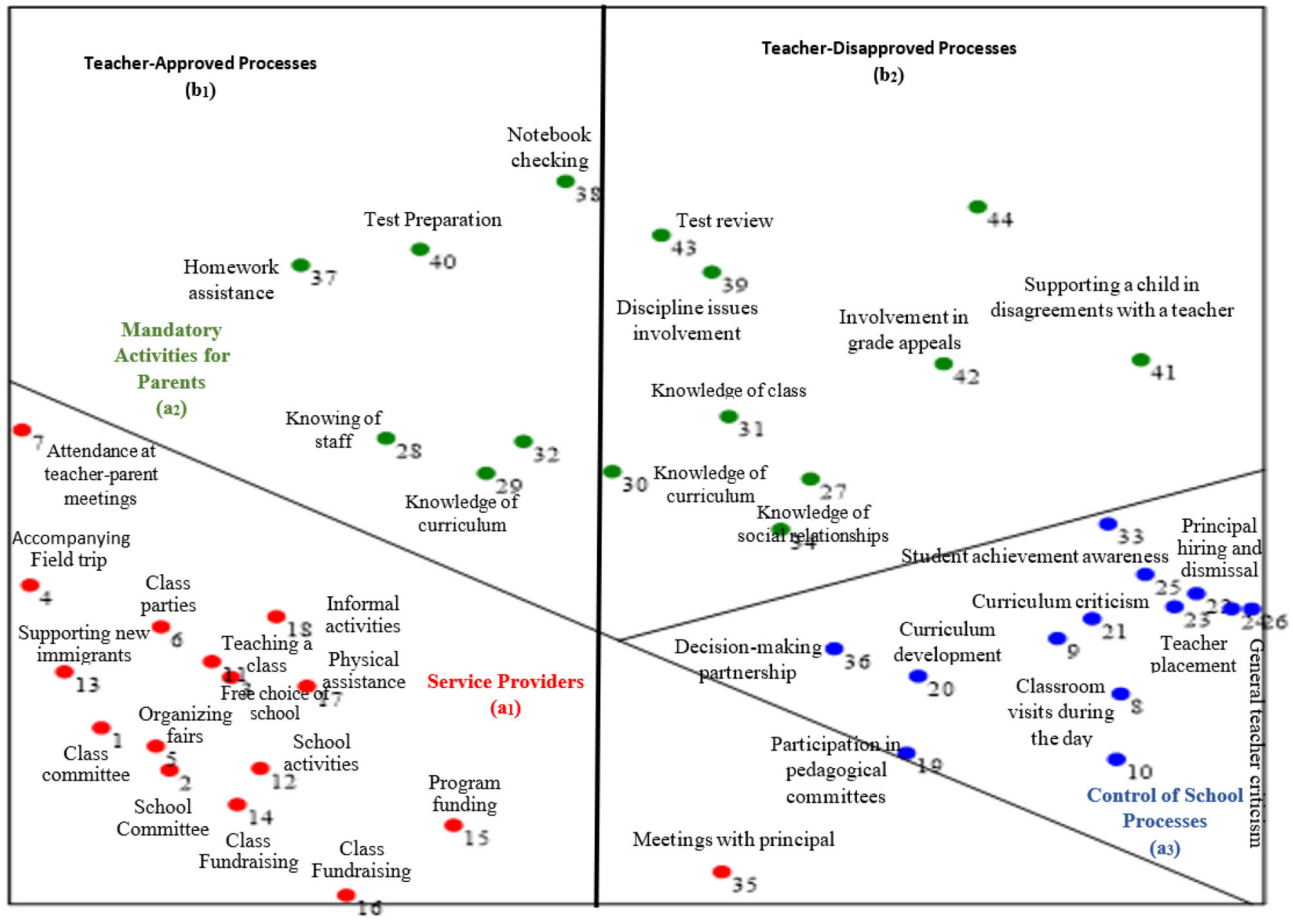
The integrated SSA map – teachers' definition parental involvement.

Facet A in the teachers' map creates an angular data distribution. Examining the map (see Figure 4) reveals that it is divided into three angular regions. However, the map is split into two parts: an upper section containing two regions and a lower section containing one region. The upper region contains 15 items expressing activities that teachers consider “mandatory” for every parent by virtue of being a parent (a_2_). This region represents topics and school-related tasks that parents are required to perform. Parents are responsible for their children and thus for these matters relating to their children and the school (for example, “involvement in disciplinary issues”—item 39; “help with exam preparation”—item 40).

The central right region contains 13 items expressing process control (a_3_). This region is characterized by areas related to parental oversight of school processes, including supervision of the educational staff. Through information obtained from these activities, parents can know what is happening in the school and even provide criticism directed at the school's educational staff and external factors (for example, “hiring and firing administrators”—item 23; “curriculum development”—item 20).

The left region contains 16 items representing the expression of “service providers” (a_1_). This region represents processes in which parents take an active part for the benefit of the school and provide services that help the educational staff in ongoing activities; parents are viewed by the teachers as “service providers,” responding to school needs when required (for example, “helping with class parties”—item 6; “fundraising for the school”—item 16).

## 4 Discussion

For decades, “parental involvement” has been extensively researched worldwide (Oostdam and Hooge, 2013; Friedman, 2011; Lavenda, 2011; Goldring, 1990; Feuerstein, 2000). However, there is no consensus regarding its precise definition (Fisher, 2018). While separate examinations of teachers' and parents' perspectives exist, comparative analyses of parents' vs. teachers' perceptions of this concept are notably scarce in the professional literature. Hence, this research focused on understanding the different perceptions of teachers and parents regarding “parental involvement.” The findings indicate that parents and teachers agree on several aspects of parental involvement; however, a deeper analysis reveals that parents' and teachers' conceptualizations of “parental involvement” are not entirely identical.

During initial data processing and confirmatory factor analysis, distinct factors emerged for the two population groups despite identical statements (except for several items excluded from the teacher population sample, as detailed in Section 3). The researchers viewed this as a first “red flag,” suggesting that analyzing the data through facet theory would shed light on these differences. Given that one of the central tools in this approach is the “mapping sentence,” two different mapping sentences were formulated: one for the teacher population and another for non-teacher parents. The spatial visualization of SSA maps and their comparison illuminates the fundamental differences between these populations.

The first distinction between teachers' and parents' perceptions was evident in the initial factor division. The parents' perception encompassed forty-four activities, while the teachers' perception comprised forty-one activities. This indicates that teachers do not agree with certain statements of parental involvement. The items excluded from the teachers' perceptions revealed major differences in how each group conceptualizes parental involvement. For instance, teachers view parent-teacher meetings as routine, or even “mandatory,” rather than as a sign of involvement. Similarly, they consider meetings with principals on general matters outside the scope of appropriate parental engagement, believing that parents should primarily interface with teachers. The matter of weekly phone contact with homeroom teachers was notably opposed by teachers, leading to its exclusion from their conceptualization of parental involvement. The differences between the activities included in the teachers' and the parents' maps highlighted fundamental disparities in how each group defines and understands parental involvement.

### 4.1 Mapping sentences

According to the parents' mapping sentence, parent X perceives parental involvement as comprising four domains:

Support of School Resources—This pertains to areas where parents actively support and participate in school-related matters. It encompasses parental participation in school-wide activities, providing physical or financial support, and participating in school and class committees. In this domain, parents respond to school-defined needs rather than initiating involvement, maintaining a relatively passive role (Tauber et al., 2023).

Supervision of School Processes—In this domain, parents actively supervise all school aspects, including pedagogical matters typically managed by educational staff. The term “supervision” implies a degree of distrust in the entity supervised. This activity includes participation in pedagogical committees, curriculum development, and involvement in hiring and dismissing teachers and administrators. Thus, in this domain, parents' functions include goal-setting and decision-making (Ðurišić and Bunijevac, 2017).

Partnership in External Pedagogical Processes—This domain represents collaboration between the school and the parents. While schools maintain responsibility during school hours, parents actively partner in extending school-based activities. The research literature suggests home-school continuity is crucial for students' success across all demographic groups (Efraim, 2014). Parents view disciplinary issues as originating at school but requiring parental intervention outside the school premises.

Awareness of Internal Pedagogical Processes—“Awareness” contrasts with indifference, indicating parents' active interest in school operations and their children's education (Fisher and Friedman, 2009). At the same time, awareness contrasts with the notion of supervision and monitoring, as the findings of this study clearly indicate that awareness is not related to active participation but to parents' knowledge of various school aspects, including familiarity with educational staff, school activities, curricula, and classroom social dynamics.

The dependency focus in each process can be either:

- Parent-dependent: Parents decide whether to participate
- School-dependent: Requires school agreement and cooperation

Each item is rated on an intensity scale ranging from very high to very low dependence levels.

By contrast, according to the teachers' mapping sentence, teacher X perceives parental involvement as comprising three domains:

Supervision of School Processes—Similar to parents' perception, “supervision” carries negative connotations of distrust, suggesting parents need to “check” on teachers. Parents' function involves goal-setting and decision-making (Friedman, 2011), expressing opinions on curricula and staff employment.

Mandatory Parental Activities—Activities that teachers consider fundamental parental responsibilities, i.e., basic obligations inherent to parenthood.

Parents as “Service Providers”—Teachers view parents as providing a necessary service, encompassing areas where teachers desire and need parental involvement, such as fundraising or conducting class activities.

These domains exist on a spectrum between:

- Teacher-approved activities: Teachers welcome or desire parental involvement
- Teacher-rejected activities: Teachers oppose parental involvement

Each item is rated on an intensity scale ranging from very high to very low approval.

Examining the mapping sentences and resulting maps reveals that parents' conceptualization of parental involvement aligns more closely with recent scholarly perspectives. This contemporary view regards parents as school partners who engage in activities that benefit their children and the broader school community (Ma et al., 2016; Tauber et al., 2023).

In contrast, the teachers' perception of parental involvement reflects perspectives dating back three or more decades. There are areas where teachers are absolutely opposed to parental participation, whereas when parental involvement assists the teachers' endeavors, the teachers find this involvement entirely agreeable. In other words, their views of parental involvement are based entirely on self-interest and completely ignore the parents' interests. Thus, teachers predominantly seek parental help that does not interfere in their professional domain (Boonk et al., 2018; Ðurišić and Bunijevac, 2017).

As shown, the application of Facet Theory in this research and the resulting SSA maps facilitated our understanding of parents' and teachers' perceptions of parental involvement.

The picture emerging from parents' perspectives (see Figure 4) emphasizes the locus of control (Facet B). Parents acknowledge that their involvement cannot materialize in certain domains without the teacher's cooperation. Tauber et al. (2023) reported similar findings. Their research suggests that when schools (administrators and teaching staff) oppose parental involvement, such engagement cannot occur. In other words, while parents can initiate involvement in areas under their control, school-dependent activities require institutional cooperation.

The teachers' map (see Figure 4) differs significantly from the parents. It includes two structures: the first structure (Facet A) is polar; the second structure (Facet B) is axial with two regions (right and left). The polar structure divides the map into three regions, each representing one of the main domains teachers define as parental involvement. The axial structure presents a division wherein each region represents teachers' level of agreement with the action. The degree of agreement moves along an axis from right to left, with teacher-approved activities located on the right side, and the agreement level decreases as one moves leftward.

More precisely, activities involving potential parental involvement closer to the left axis represent disruptive actions teachers find disruptive to daily school life. To comprehend this division in-depth, we examined the items at the extremes, as these best explain the entire facet. The results reflected conceptions of parental involvement that were prevalent three decades ago (Boonk et al., 2018). For example, chaperoning field trips, located at the beginning of the agreement axis on the right side (at the map's right edge), represents a desirable activity accepted by teachers—assistance they want and need. On the left side, at the map's left edge, we found statements referring to “general criticism of teachers,” indicating their resistance to criticism. Given that teachers' current view of parental involvement coincides with past perceptions, it is understandable that the notion of accepting criticism would be foreign to them. They preferred that parents refrain from intervening in matters or actions that might challenge their professional performance. While not always explicitly stated, teachers effectively conveyed the following message: “Why should parents comment on the amount of homework I assign to my students? I am the professional, and I will make these decisions.”

We can examine select statements to better understand the fundamental differences between parents' and teachers' perceptions. The first concerns parent committee matters. Interestingly, teachers viewed “participation in class committee” (item 1, located in region a_1_/b_1_, Figure 4) as a service provision activity. The item's position near the region's edge indicates broad teacher agreement regarding this activity. In contrast, parents viewed class committee participation as supporting school resources. While this item appears in the school-dependent region of the parents' map, it is spatially positioned close to the boundary between school and parent-only decisions.

Interestingly, the Ministry of Education does not mandate class committees. The latest executive circular (Israeli Ministry of Education, 2019) does not require schools or educational staff to establish class committees; hence, none will be established if a school opposes having class committees. It appears that most parents remain unaware of this directive; if they had knowledge of the Ministry's position on the matter, this item would likely be located at the edge of the region, indicating school-dependent actions.

According to the parents' perception, “free choice of school,” positioned in region a_1_ of the map (see Figure 3), is related to resource support. Its proximity to the edge suggests that parents feel that when choosing a particular school for their children, they effectively demonstrate their support for it. This finding aligns with previous research by Ma et al. (2016), which explained that when parents choose and support a school, they also want to participate in and impact school affairs.

Conversely, in the teachers' map (see Figure 4), this item appears in region a_1_, which refers to parents as “service providers.” This indicates that teachers view parents' school choice as providing a service for them, presumably under the following assumption: “If you chose us, you trust us, so let us do our job while you cooperate and do as we ask.” Placing school choice in the “service provider” region of the map aligns with findings from Barger et al. (2019), which describe parents as mere service providers who do not influence school-related domains. The difference between the parent and teacher maps in terms of the placement of this item demonstrates their significantly divergent perceptions. Parents see school choice as a pathway to partnership, while teachers view it as a means to prevent parental “interference” and secure exclusive authority over school operations.

A similar pattern regarding parents' desire for greater involvement by virtue of their support can be seen at the boundary between region a_1_ in the parents' map and the supervision area. Here, two very similar items are positioned: “project funding” and “school fundraising.” These items fall within the resource support region but spatially are located closest to the “supervision” region. This highlights the proximity between financial support and school supervision, suggesting that when parents provide support, they also wish to supervise and participate in additional school processes.

These findings are supported by another recent study, which demonstrated that financial investment by parents leads to increased demands on schools and education authorities (Ðurišić and Bunijevac, 2017). Again, in contrast to the parents, who view financial assistance as a pathway to partnership and sometimes supervision, in the teachers' map, these items are positioned at the edge of region a_1_ and are viewed by teachers as part of the services parents provide.

Examining region a_2_ (see Figure 3) in the parents' map, which refers to “supervising school processes,” two extreme items are particularly prominent. One is “meetings with the principal,” which lies on the boundary between “supervising school processes” and “support of school resources,” indicating a lack of consensus regarding the category to which it belongs. Most Parents view meetings with the principal as part of monitoring school processes, though some consider them a form of resource support. In the latter case, parents likely view their role as maintaining and promoting existing conditions rather than overseeing school operations. As mentioned, this item is absent from the teachers' map. It was eliminated during the initial factor division, indicating that teachers do not perceive this item as part of the integrated actions that constitute “parental involvement.”

The second extreme item is “expressing opinions about homework quantity” (item 10 on the extreme right side of region a_2_ in Figure 3 of the parents' map), which parents believe is at the heart of educational process supervision. Having the broadest parental consensus suggests that parents view this item as the most fundamental type of supervision. In the teachers' map (Figure 4), this item (opinion on homework quantity) also appears in supervision region a_3_, indicating similarity in teachers' and parents' perceptions regarding this item, whereby both groups view the expression of opinions about homework quantity as a supervisory element. However, the gap between teachers' and parents' perceptions is evident in the axial deployment. This item is positioned in the region that corresponds with actions that teachers disapprove of because they view parents' interference regarding homework quantity as undermining their professionalism and are unwilling to accept parental input on this matter. Parents, conversely, see this action as an essential part of parental involvement and, as mentioned, as the most fundamental form of supervision and monitoring.

Parents view their supervision of school processes as vital, whether it takes place within or beyond the school boundaries. They consider it essential to “support the child when disagreeing with the teacher” (item 41 in region a_3_ of the parents' map, Figure 3), especially because the parents are not present within school walls when such events occur. Although parents cannot control these events, they recognize that supporting their children is both a necessity and an obligation.

In the teachers' map (Figure 4), this item (41) appears in the right-hand side of region a_2_, which teachers classify as “mandatory parental activities.” This suggests a quasi-agreement between parents and teachers regarding parents' duty to support their children. However, teachers still view such support as closely linked to parents' supervisory mechanisms toward the school. Moreover, the item is positioned at the extreme right of teacher disapproval region, indicating that while teachers understand this to be an inherent part of parental involvement, they would prefer that this action did not occur.

In conclusion, we can say that parents have expanded their perceptions of parental involvement (in comparison with their past perceptions). Their current views are progressive, and their definitions align with those proposed in the recent research literature (Boonk et al., 2018; Tauber et al., 2023). Parents believe that as society becomes more involved across all dimensions, their involvement and partnership in the education system must figure prominently. This is not a marginal process that can be sidelined according to school staff preferences. In contrast, teachers still prefer parents to provide nothing more than “auxiliary help,” assisting the teachers when asked to do so but otherwise refraining from interfering in matters pertaining to their professional performance. The teachers apparently have failed to recognize the profound social processes that have affected parents' position within the educational establishment over the past three decades.

**Figure 5:**
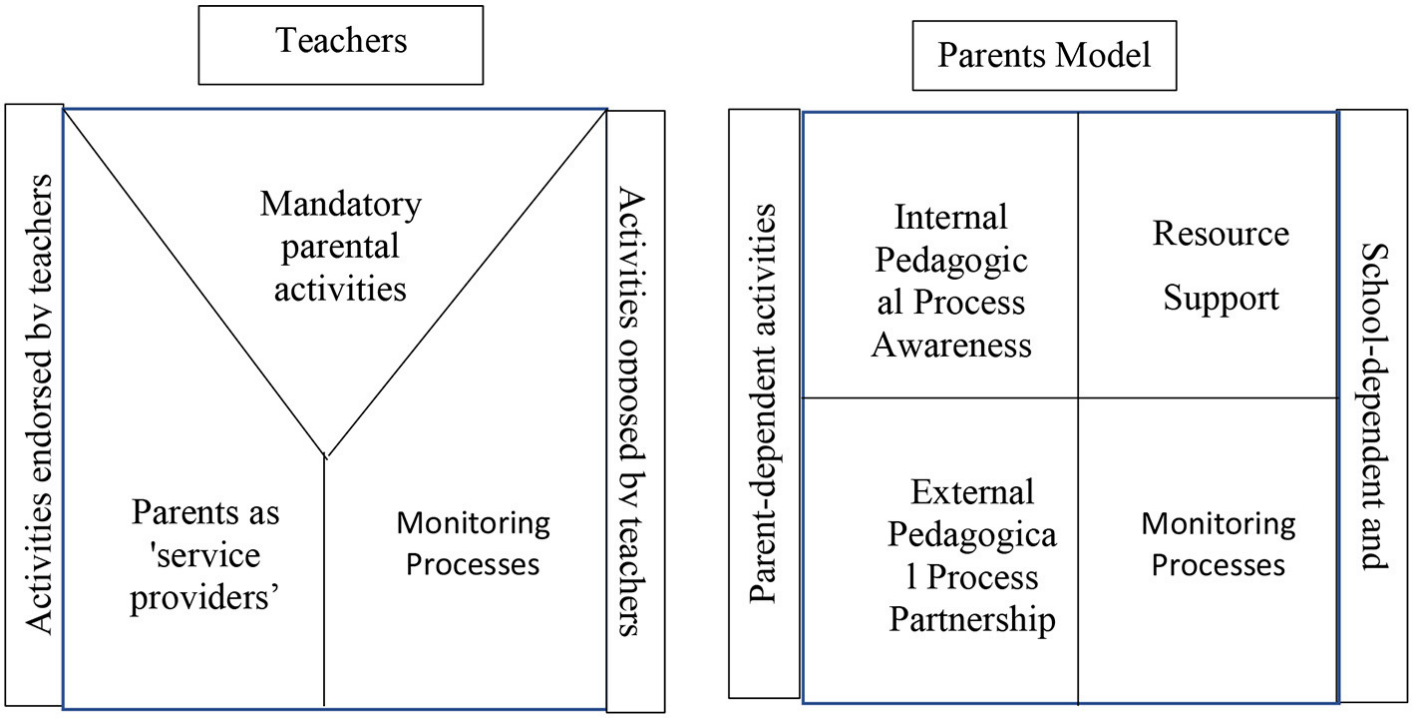
Models of perceived involvement: parents and teachers.

Indeed, Facet Theory has enabled us to construct two models: the parental involvement model from the parents' perspective and the parental involvement model from the teachers' perspective. These two models represent two perceptions and two worlds of thinking and vision, which are summarized in Figure 5.

### 4.2 Research contribution

Studies examining parental involvement consistently demonstrate its importance, providing evidence that parental involvement enhances students' academic achievement, improves their behavior within school, and serves as an indicator of school excellence. Therefore, reaching consensus on the meaning of “parental involvement” is particularly crucial. When gaps exist, as found in this research, the model helps each “stakeholder” better understand the other's perspective.

Only when one party understands the language spoken by the other can both parties engage in meaningful roundtable discussions to resolve fundamental disagreements and find a bridge to connect the two perspectives. Such dialogue can foster a culture of positive involvement and authentic communication between both parties, with the clear understanding that both sides fundamentally desire the child's best interests.

Additionally, this research aids in conceptualizing the perspectives of both teacher and parent populations separately. Such conceptualization can significantly contribute to developing mutually agreed-upon policies for parental participation in the education system on one hand and better teacher engagement and partnership on the other. These understandings, combined with developing frameworks of successful partnerships and processes in schools based on community-school collaboration, can lead to enhanced work practices between teachers, school administration, and parents, thereby improving schools overall.

### 4.3 Study limitations

This study has several important limitations that must be acknowledged when interpreting the findings:

1. Sample Representativeness:
  - The parent sample exhibits significant educational bias, with 83.3% holding academic degrees, compared to 27.3% of Israeli women nationally (OECD data). This overrepresentation of highly educated parents likely reflects individuals who are more engaged with educational matters and may limit generalizability to the broader Israeli parent population.
  - Gender distribution: The second most significant limitation concerns the representativeness of the parent sample. With 86% female participants, extensive literature consistently shows that women demonstrate higher levels of school engagement and are more likely to participate in educational activities and communication with schools (Kim, 2022; Wei et al., 2019). Therefore, the higher female participation rate in this study may reflect authentic patterns of parental involvement rather than sampling bias alone. Mothers are more likely to attend parent-teacher conferences, participate in school committees, volunteer for school activities, and engage in home-school communication.
2. Sampling methodology: The online distribution method precluded the calculation of an accurate response rate, as the total number of individuals who received the questionnaire could not be determined. Digital dissemination through multiple channels made tracking the survey reach impossible, with data available only for completed responses (*N* = 430).
3. Temporal context: Data collection during the 2020–2021 academic year occurred during the COVID-19 pandemic, which significantly disrupted educational delivery and may have influenced both parents' and teachers' perceptions of involvement due to increased home-school collaboration needs and remote learning requirements.
4. Cultural and educational context: The findings are specific to the Israeli educational system and may not apply to other cultural contexts with different parent-school relationship norms, educational structures, or cultural expectations regarding parental involvement.
5. Teacher sample characteristics: The teacher sample, although more balanced in terms of educational background, was heavily female (93.5%), which may not capture the full range of teacher perspectives across different school types and teaching contexts.

These limitations require cautious interpretation when generalizing findings to broader populations. Future research should employ more representative sampling methods to ensure demographic balance.

## Data Availability

The raw data supporting the conclusions of this article will be made available by the authors, without undue reservation.

## Footnotes

^1^See explanation of “awareness” on page 3.

^2^The Coefficient of Alienation is a technical metric that indicates the extent to which the physical distances between items in the obtained SSA (Smallest Space Analysis) map accurately reflect their correlation matrix. Specifically, it quantifies how successfully the HUDAP (Hebrew University Data Analysis Program) software is.

^3^The Separation Index (SI) is a metric that quantifies the degree to which the obtained empirical structure reflects the hypothesized content domains in the mapping statement. It indicates the goodness of fit between the theoretical model under examination and the spatial distribution of empirical data points in the resulting map. The Separation Index accounts for both the number of items that “deviate” from their hypothesized regions and the distance of these items from their hypothesized areas. The index is computed based on the sum of distances between each point's actual location on the map and its hypothesized position. The range of the Separation Index values falls between 0 and 1, where higher values indicate better fit.

^4^The Coefficient of Alienation is a technical metric that indicates the extent to which the physical distances between items in the obtained SSA (Smallest Space Analysis) map accurately reflect their correlation matrix. Specifically, it quantifies how successfully the HUDAP (Hebrew University Data Analysis Program) software has optimized the spatial arrangement of items. The Coefficient of Alienation ranges from 0 to 1, with lower values indicating superior goodness of fit. A coefficient value of 0.20 or lower demonstrates good correspondence between the inter-item distances on the map and their correlations.
